# Ternary CNTs@TiO_2_/CoO Nanotube Composites: Improved Anode Materials for High Performance Lithium Ion Batteries

**DOI:** 10.3390/ma10060678

**Published:** 2017-06-20

**Authors:** Mahmoud Madian, Raghunandan Ummethala, Ahmed Osama Abo El Naga, Nahla Ismail, Mark Hermann Rümmeli, Alexander Eychmüller, Lars Giebeler

**Affiliations:** 1 Leibniz-Institute for Solid State and Materials Research (IFW) Dresden e.V., Institute for Complex Materials, Helmholtzstr. 20, D-01069 Dresden, Germany; raghu.ummethala@gmail.com (R.U.); M.Ruemmeli@ifw-dresden.de (M.H.R.); l.giebeler@ifw-dresden.de (L.G.); 2 Technische Universität Dresden, Physical Chemistry, Bergstr. 66b, D-01069 Dresden, Germany; alexander.eychmueller@chemie.tu-dresden.de; 3 National Research Centre, Physical Chemistry Department, 33 El-Buhouth St., Dokki, EG-12622 Giza, Egypt; nahlaismail24@yahoo.com; 4 Egyptian Petroleum Research Institute, Catalysis Department, Refining Division, Nasr City, EG-11727 Cairo, Egypt; amo_epri@yahoo.com; 5 College of Physics, Optoelectronics and Energy & Collaborative Innovation Center of Suzhou Nano Science and Technology, Soochow University, CN-215006 Suzhou, China; 6 Centre of Polymer and Carbon Materials, Polish Academy of Sciences, ul. M. Curie-Skłodowskiej 34, PL-41-819 Zabrze, Poland

**Keywords:** titanium dioxide, cobalt oxide, anodic oxidation, spray pyrolysis, carbon nanotubes, mixed oxide nanotubes, composite materials

## Abstract

TiO_2_ nanotubes (NTs) synthesized by electrochemical anodization are discussed as very promising anodes for lithium ion batteries, owing to their high structural stability, high surface area, safety, and low production cost. However, their poor electronic conductivity and low Li^+^ ion diffusivity are the main drawbacks that prevent them from achieving high electrochemical performance. Herein, we report the fabrication of a novel ternary carbon nanotubes (CNTs)@TiO_2_/CoO nanotubes composite by a two-step synthesis method. The preparation includes an initial anodic fabrication of well-ordered TiO_2_/CoO NTs from a Ti-Co alloy, followed by growing of CNTs horizontally on the top of the oxide films using a simple spray pyrolysis technique. The unique 1D structure of such a hybrid nanostructure with the inclusion of CNTs demonstrates significantly enhanced areal capacity and rate performances compared to pure TiO_2_ and TiO_2_/CoO NTs, without CNTs tested under identical conditions. The findings reveal that CNTs provide a highly conductive network that improves Li^+^ ion diffusivity, promoting a strongly favored lithium insertion into the TiO_2_/CoO NT framework, and hence resulting in high capacity and an extremely reproducible high rate capability.

## 1. Introduction

Electrochemical performance of pure TiO_2_ can be improved by mixing them mechanically with some carbon derivatives such as graphene, carbon black, or CNTs during a slurry preparation [1,2,3]. Unfortunately, it is not possible to use the mechanical mixing for TiO_2_ NT arrays, as this technique destroys their tubular structure. Another alternative has been oriented to the thermal treatment of anodically synthesized TiO_2_ NTs in a methane–hydrogen-containing atmosphere in the presence of Fe precursors to perform carbon coating. Such carbon-coated TiO_2_ NTs showed a remarkable improvement in the electrical conductivity when tested for supercapacitor applications [4]. CNTs are very attractive materials for synthesizing elegant heterojunction composite anodes with TiO_2_ NTs, owing to their high electronic conductivity, structural stability, and the ease of manufacturing through mass production routes [5,6,7,8].

Other previous studies address the synthesis of many binary composites from CNTs and pure TiO_2_ for lithium ion batteries. The prepared TiO_2_ commonly exhibits forms like nanoparticles or nanospheres, which require additional polymeric binder and conductive carbon in the electrode manufacturing [6,7,8,9,10,11]. Additionally, most of these studies show that CNTs are utilized as a support for TiO_2_ (or TiO_2_ is filled into spacings between the CNTs in a CNT array).

In the present study, we propose a new strategy to realize deposition of CNTs on anodically fabricated TiO_2_/CoO NTs using a simple spray pyrolysis technique. CNTs function as conductive networks that connect TiO_2_ nanotubes from the opposite side of the current collector, allowing for better electrical and ionic conductivities, and paving the way for superior lithium ion insertion with excellent rate performance. In addition, the as-fabricated ternary composite is directly used as a binder- and additive-free electrode, taking advantage of utilizing the alloy substrate as a current collector and recommending them for low cost and high performance batteries.

To the best of our knowledge, our study is the first attempt to fabricate a ternary composite electrode by applying surface modifications on anodically grown TiO_2_/CoO nanotube arrays with CNTs for lithium ion battery applications. We believe that integrating such a ternary composite in interdisciplinary applications will open the door for achieving new advancements (which will also apply to a wide range of other subjects, such as water purification, water splitting, photocatalysis, and supercapacitors).

## 2. Experimental

### 2.1. Synthesis of TiO_2_ and TiO_2_/CoO Nanotubes

TiO_2_/CoO NTs were grown on Ti_80_Co_20_ alloy substrates using anodic oxidation technique, in which a formamide-based solution containing 0.3 M NH_4_F (99% purity, Merck, Darmstadt, Germany) and 0.1 M H_3_PO_4_ (85%, Merck, Darmstadt, Germany) was utilized as an electrolyte, while the formation voltage was adjusted to 60 V for 10 h. The formation potential (60 V) was chosen based on the highest areal capacity achieved by such nanotubes, which is related to the highest loading mass of the electrodes formed between 20 and 60 V [12]. The detailed preparation information of the alloy substrate and fabrication procedures of TiO_2_/CoO NTs are described in our previous study [12]. As a reference, pure TiO_2_ nanotubes were synthesized from Ti metal substrates (0.25 mm thickness, 99.8% purity, Alfa Aesar, Karlsruhe, Germany), employing the same anodization conditions. The as-prepared TiO_2_/CoO NTs were transferred into the spray pyrolysis system for the deposition of CNTs.

### 2.2. Synthesis of CNTs@TiO_2_/CoO NT Composite

Multi-walled CNTs were synthesized on the surface of TiO_2_/CoO NT arrays using a single-step spray pyrolysis technique. A mixture of 1.25 g ferrocene (C_10_H_10_Fe, 99.5% purity, Alfa Aesar, Karlsruhe, Germany) and 0.5 g benzeneboronic acid (C_6_H_7_BO_2_, 98% purity, Alfa Aesar, Karlsruhe, Germany) in 100 mL toluene (C_7_H_8_, 99.99% purity, Merck, Darmstadt, Germany) was used as precursor solution. A steady precursor spray was generated with the help of a spray system and the fine spray was carried by argon gas into a horizontal quartz tube, maintained at a temperature of 860 °C. The as-prepared TiO_2_/CoO NT sample was placed in the middle of the quartz tube. The synthesis was carried out until the precursor solution was exhausted (~25 min). Fe from ferrocene nucleates the growth of CNTs, whereas toluene serves as the carbon precursor. Boron, from the benzeneboronic acid, aids in the formation of stable bends in the CNTs and significantly increases the aspect ratio of the nanotubes, resulting in a uniformly covered CNT layer on the substrates [13].

### 2.3. Synthesis of Pure TiO_2_ and CNTs@TiO_2_ NTs

In comparison, TiO_2_ nanotubes were grown on the surface of Ti foils (0.25 mm thick, 99.8% purity, Alfa Aesar, Karlsruhe, Germany), employing the same anodic oxidation conditions as used for the Ti-Co alloy. Onto the as-prepared TiO_2_ nanotubes, CNTs were covered under the same preparation conditions as used for CNTs@TiO_2_/CoO NT composites (as described in the previous section).

### 2.4. Material Characterization

The surface morphology of the as-prepared TiO_2_/CoO and CNTs@TiO_2_/CoO NTs was investigated by field emission scanning electron microscopy (Gemini LEO 1530, Zeiss, Oberkochen, Germany and Nova Nanosem 200, FEI Electron Optics, Eindhoven, The Netherlands) at an acceleration voltage of 20 kV. Phase analyses of the as-fabricated TiO_2_ and TiO_2_/CoO nanotubes (before and after the deposition of CNTs) were performed by X-ray diffraction (XRD, X’Pert Pro, PANalytical, Eindhoven, The Netherlands), using Co Kα radiation and a PIXcel detector in Bragg-Brentano geometry. Raman spectra of the as-fabricated CNTs@TiO_2_/CoO NTs were recorded at a laser power of 8 mW and an excitation wavelength of 532 nm (DXR Smart Raman, Thermo Scientific, Madison, WI, USA). STEM-EDXS was carried out for the as-prepared TiO_2_/CoO NTs using a FEI Tecnai F30 microscope (FEI Electron Optics, Eindhoven, The Netherlands) at 300 kV acceleration voltage.

### 2.5. Coin Cell Assembly and Electrochemical Testing

We employed the as-prepared TiO_2_/CoO and CNTs@TiO_2_/CoO NTs directly, without additional binder or conductive additives taking advantage of utilizing the Ti-Co substrates as current collectors. Half cells were assembled as coin cells of the CR2025 type in an Ar-filled glove box under controlled O_2_ and H_2_O content (<0.1 ppm), in which the CNTs@TiO_2_/CoO NTs were used as working electrodes, and a lithium foil (Alfa Aesar, Karlsruhe, Germany, 99.9%) as a counter electrode; a Celgard 2500 polypropylene separator (Celgard, Charlotte, NC, USA) with 16 mm diameter and 25 µm thickness was also used, along with the standard LP30 electrolyte (1 M LiPF_6_, 1:1 v/v DMC/EC, BASF Battery Materials, Independence, OH, USA). The average weight of the pure TiO_2_ and TiO_2_/CoO was determined after separating the nanotubes from Ti and Ti-Co alloy substrates by sonication in a mixture of ethanol and deionized water (volume ratio 9:1) [12]. The mass of each electrode was ≈1.066 mg. The mass of the deposited CNTs was determined by weighing the electrode before and after the spray pyrolysis step. The average CNT mass per electrode area was ≈0.12 mg. The electrochemical measurements were performed by a multichannel potentiostat–galvanostat (VMP3, Bio-Logic SAS, Seyssinet-Pariset, France). Cyclic voltammetry tests (CV) were carried out in a potential range of 0.1–3 V vs. Li/Li^+^, at a scan rate of 0.1 mV·s^−1^. The assembled cells were then galvanostatically cycled at a current density of 50 µA·cm^−2^, corresponding to a current density of 1 C between 0.1 and 3 V vs. Li/Li^+^. In comparison, TiO_2_/CoO NTs were assembled and tested under identical conditions. Electrochemical impedance spectroscopic (EIS) tests were conducted between 100 kHz and 0.1 Hz at a potential of 1.7 V after 50 charging/discharging cycles (at a current density of 50 µA·cm^−2^).

## 3. Results and Discussion

### 3.1. Characterization

**Figure 1:**
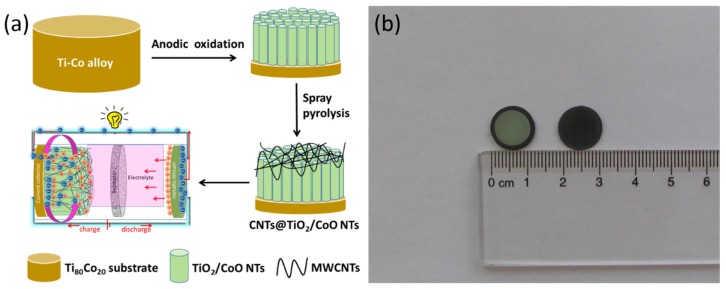
Schematic illustration of the fabrication strategy of ternary CNTs@TiO_2_/CoO NTs (**a**); a photograph of the TiO_2_/CoO NTs before (left electrode) and after CNT covering (right electrode) (**b**).

The typical fabrication process of CNTs@TiO_2_/CoO NTs is presented in Figure 1a. Firstly, the TiO_2_/CoO NTs were grown on the two-phase Ti_80_Co_20_ alloy. The as-grown TiO_2_/CoO NTs were subsequently subjected to a surface modification with CNTs through spray pyrolysis. The as-formed CNTs@TiO_2_/CoO NTs were then assembled and tested as anodes against lithium in a coin cell battery. A photograph of the pristine and CNT-covered electrode is shown in Figure 1b.

The surface morphologies of the as-formed TiO_2_/CoO NTs and post-growth CNTs were examined using SEM.

**Figure 2:**
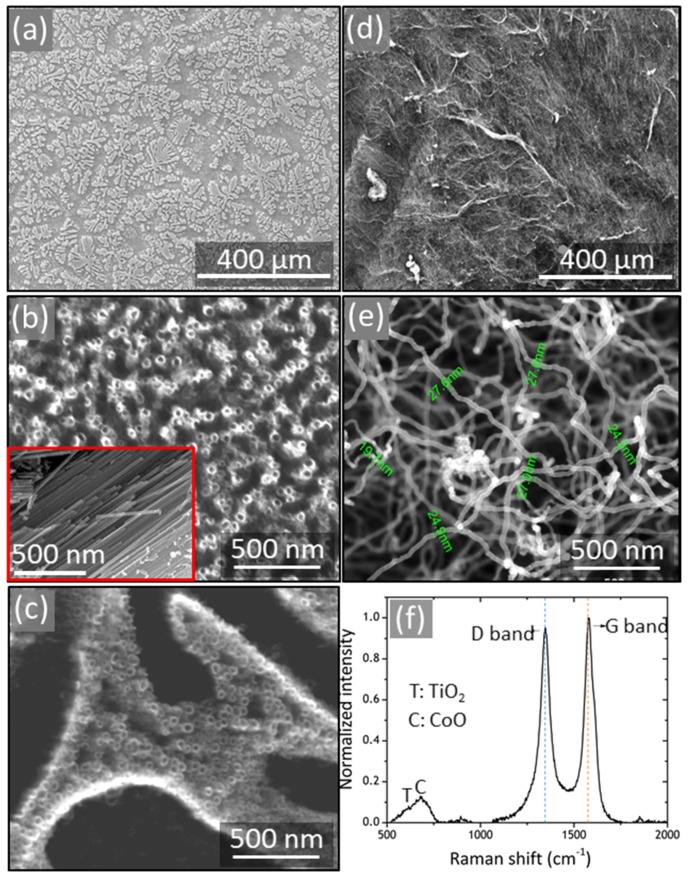
SEM micrographs, an overview of anodized Ti-Co alloy (**a**); high magnification of the *β*-Ti (**b**) and the Ti_2_Co (**c**) phases anodized at 60 V; low (**d**) and high (**e**) magnifications of the ternary CNTs@TiO_2_/CoO NTs; and Raman spectra of ternary CNTs@TiO_2_/CoO NTs (**f**).

Figure 2a shows a low magnification view of the alloy substrate after the anodization process, (where the dark and bright areas represent *β*-Ti and Ti_2_Co phases, respectively, of the Ti-Co alloy). The high magnification top view of *β*-Ti and Ti_2_Co are displayed in micrographs (b) and (c) of Figure 2. Evidently, well-ordered, clear-cut TiO_2_/CoO NTs are successfully formed on the entire surface of the Ti-Co alloy. The average diameter of the nanotubes grown on *β*-Ti is 40 nm. The nanotubes grown on the Ti_2_Co phase showed an average diameter of 37 nm. The tubular structure of the oxide film is also indicated from the cross-sectional view of the oxide layer, presented in the inset of image (Figure 2b). STEM-EDXS measurements shown in Figure S1 (Supplementary Information) reveal that the fabricated nanotubes are composed of Ti and Co oxides, implying the good mixing of the Co–O and Ti–O species in the framework (further characterization details of TiO_2_/CoO NTs are addressed in our previous report [12]). Panel (d) in Figure 2 manifests the entire overview of the oxide surface after performing the CNT growth. It clearly shows that the CNT layer covers the whole surface of the oxide NT array, indicating a uniform coverage. Figure 2e demonstrates the high magnification top-view of the as-fabricated CNTs@TiO_2_/CoO NTs sample. The outer nanotube diameter of the CNTs ranges from 20 to 27 nm (Figure 2e), which is a characteristic feature of the multi-walled carbon nanotubes grown by this technique [13]. It obviously shows that the CNTs are formed horizontally in an interwoven web-like structure, suggesting that CNTs can effectively connect the individual TiO_2_/CoO nanotubes through a high conductive network and may serve as a rather charge collector. Thus, the electrical and the ionic conductivities of the CNTs@TiO_2_/CoO NT electrode are expected to be improved allowing for a higher Li^+^ ion diffusion and storage efficiency. Successful CNTs deposition on pure TiO_2_ was also revealed from SEM micrographs in Figure S2 (Supplementary Information).

Raman spectroscopy provides important information about the carbon species, such as details related to the C-C bonds and defects that can be effectively obtained from the change in the signal shift. Figure 2f displays the Raman spectrum of the CNTs@TiO_2_/CoO NTs sample. The typical disorder-induced D band (~1335 cm^−1^) and tangential modes (G band, ~1587 cm^−1^) are observed. The D band indicates the presence of defects in sp^2^ hybridized carbon, while the G band is a characteristic of the in-plane vibration mode of sp^2^-bonded carbons. The recorded D and G values are in accordance with those previously reported for CNTs/TiO_2_ composites [6,14,15]. It is interesting to point out that a small shift to higher values was observed for both D and G bands compared to the Raman characteristics of pure CNTs [14,15,16], revealing the possible interaction between the CNTs and TiO_2_ [14,15]. Notably, a broad peak centered with its maximum at 680 cm^−1^ is observed in the spectra. This peak matches very well with the reported A_1g_ vibration mode of CoO species [17]. Additionally, a peak hump is noticed at 610 cm^−1^, which is assigned to rutile [18]. The small peak that appeared at 892 cm^−1^ may possibly be related to C–C or C–O stretching vibrations of organic carbon traces that originate from the electrolyte used for the anodic oxidation [19,20]. In conclusion, the Raman investigation confirms the successful deposition of CNTs on the TiO_2_/CoO NT surface.

**Figure 3:**
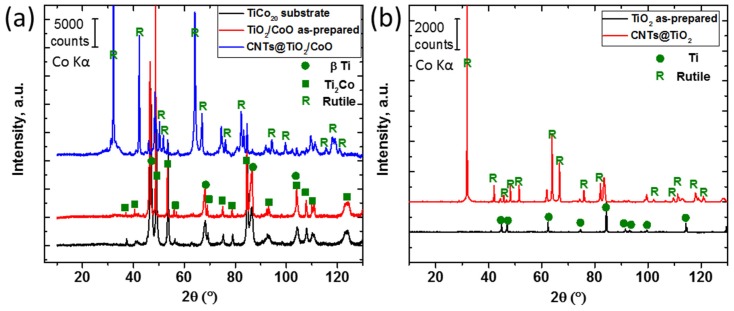
XRD patterns of the Ti_80_Co_20_ alloy substrate, the as-anodized TiO_2_/CoO NTs, and the as-prepared CNTs@TiO_2_/CoO NTs (**a**); diffractograms of the as-anodized TiO_2_ and CNT@TiO_2_ NTs samples (**b**).

XRD analysis of the TiCo_20_ alloy in Figure 3a showed reflections in good agreement with those reported for both the *β*-Ti [21] and Ti_2_Co [22] phases. No or hardly detectable reflections are further observed in the as-anodized TiO_2_/CoO sample, indicating an amorphous state of the prepared nanotubes. Also, reflections for Ti metal can only be distinguished for the as-prepared TiO_2_ NTs (Figure 3b), proving a similar amorphous nature of such nanotubes. Sharp reflections are clearly shown in the diffractograms of both CNTs@TiO_2_/CoO NTs (Figure 3a) and CNT@TiO_2_ NTs samples, which are in accordance with the Bragg positions of rutile [23]. It is known from literature [24] that rutile is obtained at a temperature higher than 800 °C, which matches with the temperature required for spray pyrolysis to form CNTs.

### 3.2. Electrochemical Testing

**Figure 4:**
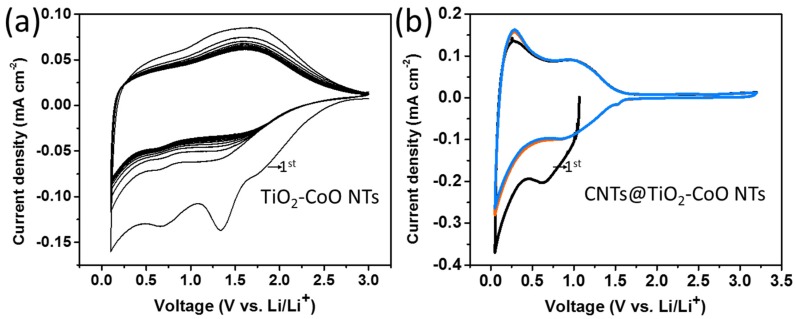
Cyclic voltammograms of TiO_2_/CoO nanotubes (**a**) and CNTs@TiO_2_/CoO NTs (**b**), measured at scan rates of 0.1 mV·s^−1^ vs. Li/Li^+^.

The electrochemical characteristics of bare TiO_2_/CoO and CNTs@TiO_2_/CoO NT nanocomposite electrodes were investigated in order to underline the synergetic effect of CNTs in the composite backbone. Since the lithium ions intercalate the CNTs at a potential lower than 1 V, the cells were measured between 0.1 and 3 V. Figure 4a shows typical CV curves obtained at a scan rate of 0.1 mV s^−1^ of a pure TiO_2_/CoO NT electrode, without CNTs. The recorded cyclic voltammograms of the TiO_2_ (shown in Figure S3, Supplementary Information) and TiO_2_/CoO NTs are, in general, consistent with previous studies suggesting a similar intercalation behavior [25,26,27]. Two broad signals are observed in the anodic and cathodic branches at 1.6 and 1.7 V vs. Li/Li^+^, respectively. This broadening in the anodic and cathodic sweeps is a special characteristic for an amorphous state of TiO_2_ and TiO_2_, mixed with another transition metal oxide [25,26]. No or hardly distinguishable signals are further detected in the following cycles:

These results indicate that the electrochemical insertion/removal reactions into/out of TiO_2_ and TiO_2_/CoO NT frameworks take place without phase transformation (as usually found for crystalline materials) [1,28,29]. Figure 4b depicts the CV curves of the CNTs@TiO_2_/CoO NT electrodes. The absence of sharp peaks and presence of two broad peaks in the anodic and cathodic branches at similar peak positions are consistent with the general CV behavior of rutile [30], and are attributed to a typical Li^+^ ion intercalation/deintercalation mechanism into or out of solid solution domains [30]. The corresponding anodic/cathodic signals are significantly shifted to the lower voltage of around 1 V vs. Li/Li^+^, compared to 1.6 and 1.8 V vs. Li/Li^+^ for the unmodified TiO_2_/CoO NTs (Figure 4a) and rutile [30], respectively. This shift in the peak potential is attributed to the presence of CNT layers that interconnect the TiO_2_/CoO NTs and enable high electrical conductivity (as well as a good lithium ion transport and, therewith, faster diffusion kinetics) [31]. Unlike TiO_2_/CoO NTs, no decrease in the current density of the redox signals occurs over cycling, indicating the good stability and better reversibility of the CNTs@TiO_2_/CoO NT electrode compared to the CNT-free electrode. These results already suggest that this composite electrode has a great potential as a promising anode for lithium ion batteries.

**Figure 5:**
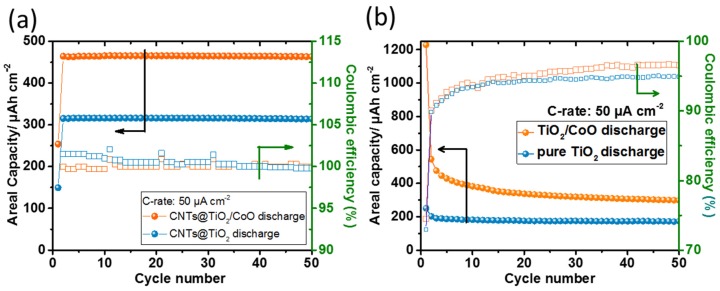
Galvanostatic areal discharging capacities as a function of cycle number, obtained at a current density of 50 µA·cm^−2^ for CNTs@TiO_2_ and CNTs@TiO_2_/CoO NT anodes and their corresponding Coulombic efficiencies (CE) (**a**); cycling performance of uncovered TiO_2_ and TiO_2_/CoO NT electrodes with the corresponding CE (**b**).

Figure 5a depicts the galvanostatic cycling performance of CNTs@TiO_2_ and CNTs@TiO_2_/CoO NT electrodes and the corresponding Coulombic efficiencies (CE) (evaluated at a current density of 50 µA·cm^−2^ between 0.01 and 3 V vs. Li/Li^+^). In the first cycle, both electrodes show a relatively low areal capacity when compared with the following cycles. This observation is in accordance with the previously described analysis of the cyclic voltammograms, where the current density in the first scan is slightly smaller (0.13 mA·cm^−2^) than those of the next sweeps (0.15 mA·cm^−2^). A possible activation of the electrode surface may be concluded to be the origin of this behavior [32].

It is noteworthy that the ternary CNTs@TiO_2_/CoO NT composite electrode exhibits an areal capacity of 460 µAh·cm^−2^, demonstrating excellent cycling stability over 50 charging/discharging cycles with a CE of ≈100%. Binary CNTs@TiO_2_ NT composite electrodes can deliver a capacity of 310 µAh·cm^−2^ (with a relatively higher CE (ca. 104%)) in the first ten cycles, reaching an efficiency of ca. 100% at the end of the 50th charging/discharging cycle. It is clear that the ternary CNTs@TiO_2_/CoO NT electrode displays around a 1.5-fold increase in capacity compared to the CNTs@TiO_2_ NT anode. Surprisingly, the binary CNTs@TiO_2_ and ternary CNTs@TiO_2_/CoO NT composites show significantly enhanced capacities, reaching up to a 1.5-fold increase in the 50th discharge cycle compared to the areal capacity of uncovered TiO_2_ and TiO_2_/CoO NT electrodes (which were tested at the same electrochemical conditions (Figure 5b)). The remarkable increase in the reversible capacity of the ternary composite is attributed to a good adhesion between the highly conductive, interlaced CNTs and the mixed oxide NTs. The boron-induced ‘elbow junctions’ of the robust CNT network further enhance the connectivity of the oxide NTs with the commonly used carbon additives, and thereby result in higher conductivities facilitating easier and faster electron transport and ion insertion/removal process. The high CE also implies a stable SEI formed on the surface of the CNTs and the oxide NTs, as well as a very low degradation of the electrolyte (due to a very low number of side reactions). As one of the components of the ternary oxide electrodes, CNTs may also contribute to the overall capacity of the cells. Hence, based on the capacity measurements conducted on the CNTs without the transition metal oxide nanotubes under identical electrochemical test conditions, we determined that the CNTs contribute only about 3.6% (16.8 µAh·cm^−2^) to the overall capacity (Figure S4, Supplementary information). Hence, the CNTs in the ternary composite electrodes mainly improve electrical connectivity and diffusion kinetics in the composite, and their storage ability can be neglected (especially when optimizing the potential window to higher potentials).

It is worth emphasizing the effect of the amorphicity and crystallinity of the TiO_2_ and TiO_2_/CoO NTs on the Li^+^ ion storage capacity at a current density of 50 µA·cm^−2^ (based on the results of the present study (Figure 5b) and our previous work) [12]. At the 50th charging/discharging cycle, the amorphous TiO_2_/CoO NTs deliver an areal capacity of 305 µAh·cm^−2^, which is higher than that found for TiO_2_/CoO NTs containing a phase mixture of crystalline anatase and rutile (280 µAh·cm^−2^). In addition, amorphous TiO_2_ NTs also show an improved areal capacity of 180 µAh·cm^−2^ compared to anatase (170 µAh·cm^−2^). The noticed enhancement in the insertion capacity of amorphous TiO_2_/CoO and TiO_2_ NTs is in accordance with previous observations, and is attributed to larger amount of disorders and defects in the amorphous structure than the crystalline one [27,33,34]. These defects offer bigger channels or more diffusion paths for Li^+^ ion migration. Nevertheless, crystallization of nanotube arrays during the CVD process carried out under 860 °C does not affect electrochemical performance, since much higher capacities are observed compared to amorphous or crystalline electrodes without a carbon coverage.

**Figure 6:**
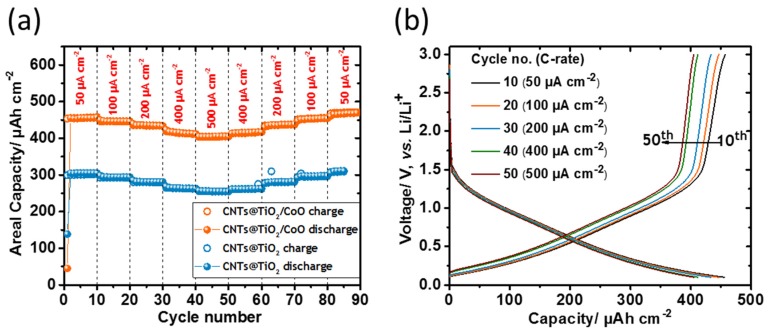
Rate capability of CNTs@TiO_2_ and CNTs@TiO_2_/CoO NT anodes (**a**); typical voltage profiles vs. Li/Li^+^ for the 10th, 20th, 30th, 40th, and 50th cycles against the areal capacity of TiO_2_/CoO NT anodes (**b**) (measured at a current rate of 50, 100, 200, 400 and 500 µA·cm^−2^, respectively).

In order to determine the electrochemical performance of the CNT-covered anodes, the electrodes were cycled at different current densities (50–500 µA·cm^−2^), which are shown in Figure 6a. The CNTs@TiO_2_/CoO NT electrode is able to deliver an areal capacity of 455 µAh·cm^−2^ when cycled at a current rate of 50 µA·cm^−2^. Only an insignificant decrease in the areal capacity is observed when the current rate is increased from 50 to 500 µA·cm^−2^, still displaying an excellent areal capacity of about 400 µAh·cm^−2^. It is crucial to note that the electrode can reversibly retain the same areal capacity even after 80 charging/discharging cycles when the current rates are gradually decreased, demonstrating the outstanding rate performance of the composite electrode. The corresponding charging/discharging voltage profiles of CNTs@TiO_2_/CoO NT anodes obtained at various current densities (50–500 µA·cm^−2^) are shown in Figure 6b. Apparently, no distinct plateaus are detected in the voltage curves matching the cyclic voltammograms discussed above (Figure 4). Similar behavior in the rate capability was also detected for CNTs@TiO_2_ NT; however, it can only reach an areal capacity of around 300 and 256 µAh·cm^−2^ at current densities of 50 and 500 µA·cm^−2^, respectively. Interestingly, the voltage profile curves of CNTs@TiO_2_/CoO NT indicate that only a small decrease in the areal capacity will be achieved if the potential window is limited to 1.5 V. In consequence, the possibility to use such materials for practical applications is underlined. These findings clearly demonstrate the effectiveness of the tightly interlaced CNT network in greatly enhancing the rate performance of the CNTs@TiO_2_ and CNTs@TiO_2_/CoO NT electrodes. Table 1 shows a comparison of capacity and reported cycles of the electrode composite described in this study, with some nanostructured TiO_2_/CNTs and CoO/CNTs composites to allow a critical rating of the new material. To the best of our knowledge ternary TiO_2_/CoO-NT/CNT systems have not been reported for battery application yet, but TiO_2_/CoO nanoparticles decorated on CNTs were designated as photocatalysts [35].

**Figure 7:**
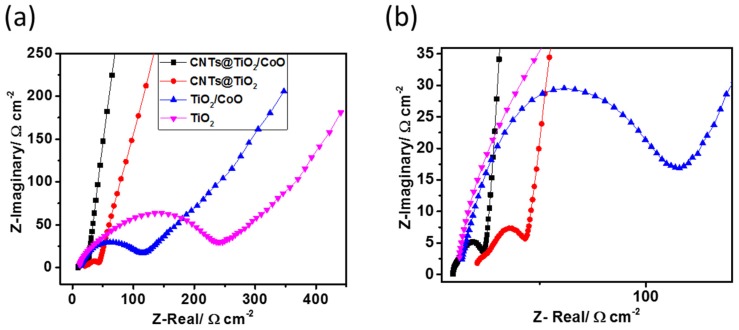
Nyquist plots of pure TiO_2_ NTs (pink triangles), TiO_2_/CoO NTs (blue triangles), TiO_2_ NTs covered with CNTs (red circles), and TiO_2_/CoO NTs covered with CNTs (black squares) (after 50 charging/discharging cycles, in the frequency range of 100 kHz to 0.1 Hz at a potential of 1.7 V vs. Li/Li^+^) (**a**); zoomed view of Figure 7a (**b**).

To gain further understanding of the effect of CNTs on the ionic conductivity of the synthesized composite anodes, electrochemical impedance spectroscopy (EIS) tests were performed for all the cells after 50 charging/discharging cycles. The Nyquist plots of all measured electrodes at 1.7 V vs. Li/Li^+^ are depicted in Figure 7a. The spectra show semicircles at high-to-medium frequency ranges, followed by inclined lines at the low frequency domain. It is known that the semicircles at high-to medium frequencies correspond to the charge transfer resistance accompanying lithium ion diffusion from the electrolyte towards the electrode/electrolyte interface [25,41,42]. The inclined lines represent lithium ion diffusion inside the electrode frameworks [12,42]. The CNTs@TiO_2_/CoO NT electrode exhibits the smallest semicircle, indicating the enhanced ionic conductivity compared to the other electrodes. Apparently, the CNT-covered electrodes show a smaller semicircle diameter than uncovered electrodes, confirming that the deposition of CNTs indeed lead to an improved ionic conductivity and, hence, enhance the electrochemical performance of lithium-ion insertion/extraction processes.

## 4. Conclusions

In our present study, we have demonstrated that a robust network of interconnected CNTs can be synthesized laterally on the surfaces of anodically grown TiO_2_ and TiO_2_/CoO nanotubes using a simple and quick, single-step, spray pyrolysis technique, leading to a successful fabrication of binary CNTs@TiO_2_ and ternary CNTs@TiO_2_/CoO nanotube composite electrodes. The electrochemical evaluation vs. the typical Li/Li^+^ reference electrode showed that the CNT-coated electrodes exhibit a 1.5-fold increase in the specific capacity compared to the uncoated anodes, along with a phenomenal rate performance between current densities of 50 and 500 µA·cm^−2^. The ternary CNTs@TiO_2_/CoO NT composite displays the best electrochemical performance among all tested electrodes. The remarkable electrochemical performance of the composite electrodes is attributed to the highly conductive CNTs interfacing the mixed TiO_2_/CoO NT framework, leading to exceptional electronic conductivity and charge transport. Secondly, the tightly interwoven CNT network may also serve as a second charge collector in conjunction with the alloy substrate, resulting in outstanding electrochemical performance. With this kind of composite electrode, it is possible to achieve excellent energy storage properties (even with electrically isolating oxide tube arrays); furthermore, we are able to significantly improve the electrical conductivity of the oxide nanotube electrodes, and thereby achieve a remarkable enhancement in their electrochemical performance (independent of the oxide morphology) by a simple surface modification step.

## Figures and Tables

**Table 1:** Performance of TiO_2_-CNTs and CoO/CNTs anode materials.

Composition	Reversible Capacity/Current Density	Given Cycles	Preparation Method	Ref.
Porous TiO_2_ nanoparticles/CNTs hybrid	200 mAh·g^−1^/100 mA·g^−1^	100	Sol-gel method	[36]
CNTs/mesoporous TiO_2_ coaxial nanocables	183 mAh·g^−1^/168 mA·g^−1^	70	Sol–gel and hydrothermal	[37]
Anatase /CNTs nanocomposite	185 mAh·g^−1^/100 mA·g^−1^	100	Hydrolysis	[7]
Mesoporous three-dimensional (3D) TiO_2_/carbon nanotube	203 mAh·g^−1^/100 mA·g^−1^	100	Solution-based synthesis process	[8]
Coaxial TiO_2_-Carbon Nanotube Sponges	210 mAh·g^−1^/100 mA·g^−1^	100	In situ hydrolysis	[5]
TiO_2_ nanoparticle-decorated carbon	190 mAh·g^−1^/100 mA·g^−1^	120	Thermal treatment	[38]
Mesoporous CoO nanorods@CNT	703–746 mAh·g^−1^/3580 mA·g^−1^	200	Hydrothermal technique	[39]
CoO nanoparticles/MWCNTs	600–550 mAh·g^−1^/15 mA·g^−1^	100	Electrophoretic deposition + CVD	[40]
CNTs@TiO_2_/CoO NT in this work	410 mAh·g^−1^/45 mA·g^−1^	50	Anodization followed by spray pyrolysis	

## Supplementary Materials

The following are available online at www.mdpi.com/1996-1944/10/6/678/s1. Figure S1: STEM image of individual nanotubes (a) and the STEM-EDXS analysis confirming that the tubes are composed of Ti and Co oxides (b,c); Figure S2: SEM images showing pure TiO_2_ nanotubes prepared at 60 V on the Ti substrate (a) and surface overview after CNT covering at different magnifications (b–d); Figure S3: Cyclic voltammograms of pure TiO_2_ nanotubes (a) and CNTs@TiO_2_ NTs, measured at scan rates of 0.1 mV·s^−1^; Figure S4: Galvanostatic cycling of CNTs foam at a current rate of 446 mA·g^−1^ between 0.1 and 3 V.
