# Supplementary material for: Ternary CNTs@TiO2/CoO Nanotube Composites: Improved Anode Materials for High Performance Lithium Ion Batteries
Source: Materials (Basel). 2017 Jun 20;10(6):678. doi: 10.3390/ma10060678 (PMC5554059; doi:10.3390/ma10060678)
Supplement: Supplementary file 1 [file materials-10-00678-s001.pdf]

Supplementary Materials: Ternary CNTs@TiO_2/CoO Nanotube Composites: Improved Anode Materials for High Performance Lithium Ion Batteries

Mahmoud Madian, Raghunandan Ummethala, Ahmed Osama Abo El Naga, Nahla Ismail, Mark Hermann Rümmeli, Alexander Eychmüller and Lars Giebeler

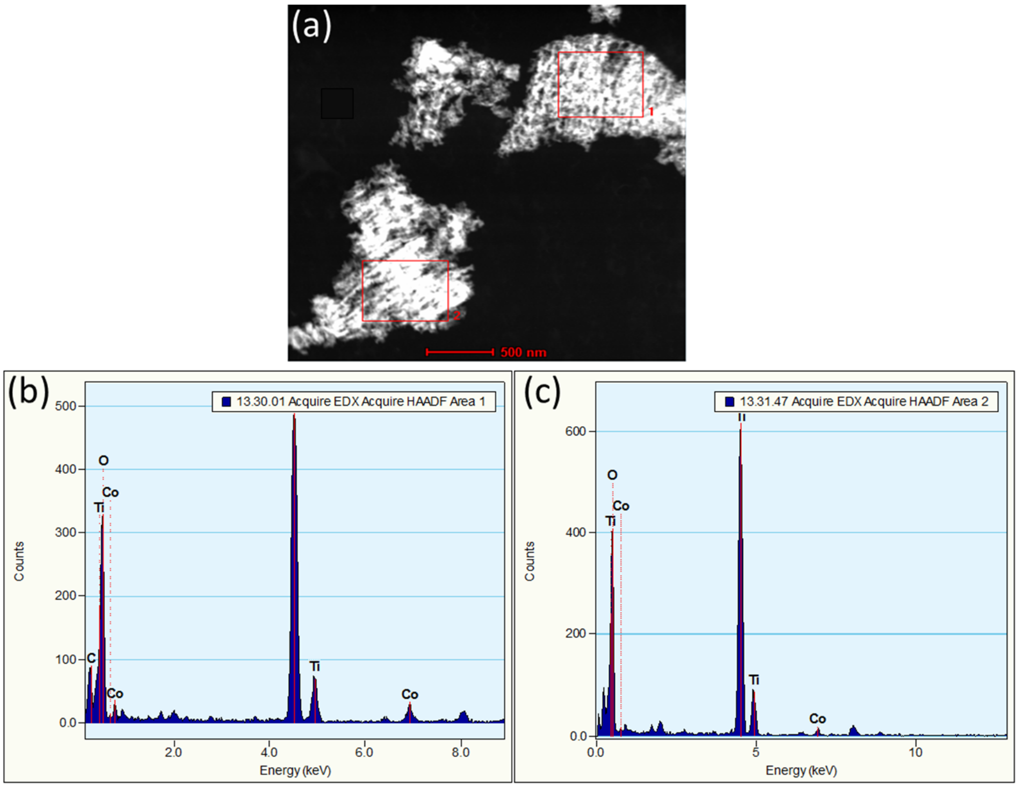

Figure S1. STEM image of individual nanotubes (**a**) and the STEM-EDXS analysis confirming that the tubes are composed of Ti and Co oxides (**b**,**c**).

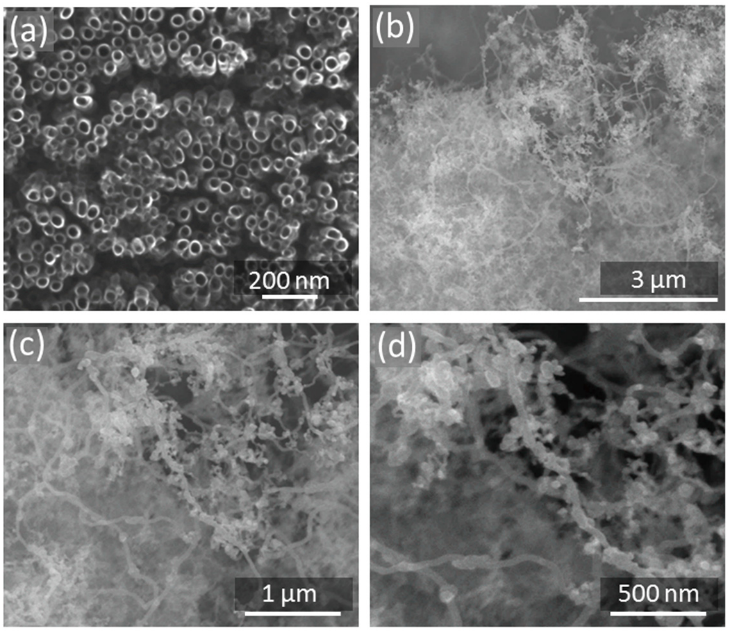

Figure S2. SEM images showing pure TiO_2 nanotubes prepared at 60 V on the Ti substrate (**a**) and surface overview after CNT covering at different magnifications (**b**–**d**).

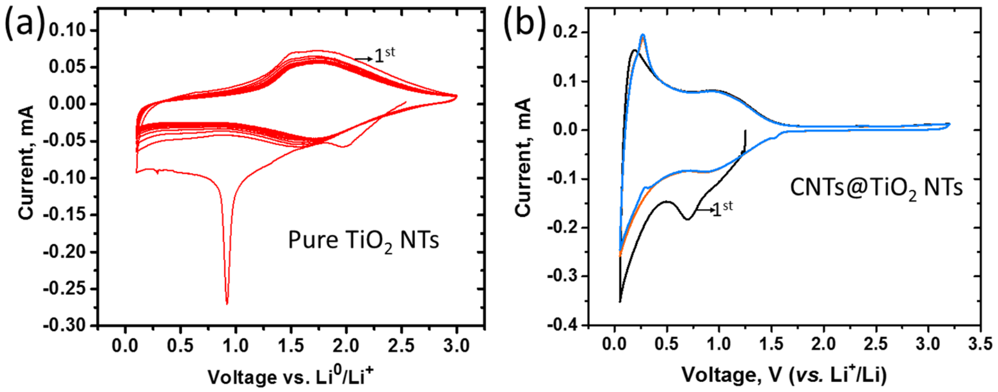

Figure S3. Cyclic voltammograms of pure TiO_2 nanotubes (**a**) and CNTs@TiO_2 NTs (**b**), measured at scan rates of 0.1 mV·s^{-1}.

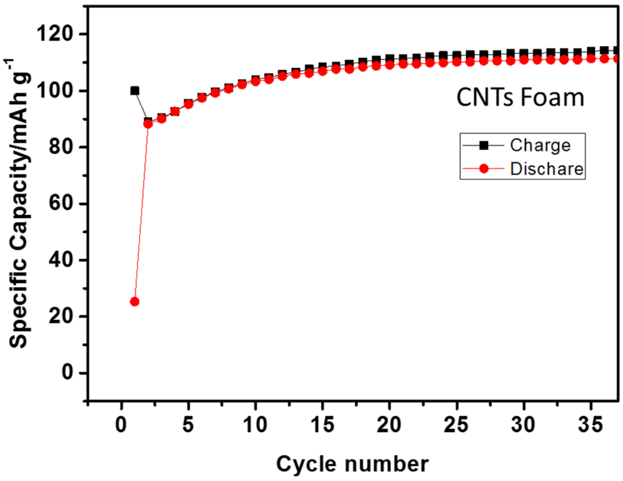

Figure S4. Galvanostatic cycling of CNTs foam at a current rate of 446 mA·g^{-1} between 0.1 and 3 V.

The mass of CNTs foam electrode was 2.730 mg. mass of TiO/CoO NT + mass of CNTs is termed as CNTs@TiO_2/CoO NT. The current density of the CNTs foam was calculated based on the average mass of CNTs@TiO_2/CoO NT (1.12 mg) that was tested at 500 μA. Therefore, the current needed for 2.730 mg CNTs is 1.218 mA, which corresponds to 446 mA·g^{-1}.

© 2017 by the authors. Submitted for possible open access publication under the terms and conditions of the Creative Commons Attribution (CC BY) license (http://creativecommons.org/licenses/by/4.0/).
